# Ca_V_3.2 T-Type Calcium Channels in Peripheral Sensory Neurons Are Important for Mibefradil-Induced Reversal of Hyperalgesia and Allodynia in Rats with Painful Diabetic Neuropathy

**DOI:** 10.1371/journal.pone.0091467

**Published:** 2014-04-04

**Authors:** Aleksandar Lj. Obradovic, Sung Mi Hwang, Joseph Scarpa, Sung Jun Hong, Slobodan M. Todorovic, Vesna Jevtovic-Todorovic

**Affiliations:** 1 Department of Anesthesiology, University of Virginia Health System, Charlottesville, Virginia, United States of America; 2 Department of Physiology, Faculty of Pharmacy University of Belgrade, Belgrade, Serbia; 3 Department of Anesthesiology and Pain Medicine, Chuncheon Sacred Heart Hospital, College of Medicine, Hallym University, Chuncheon, South Korea; 4 Department of Anesthesiology and Pain Medicine, Kangdong Sacred Heart Hospital, College of Medicine, Hallym University, Seoul, South Korea; 5 Department of Neuroscience, University of Virginia Health System, Charlottesville, Virginia, United States of America; 6 Neuroscience Graduate Program, University of Virginia Health System, Charlottesville, Virginia, United States of America; University of Waterloo, Canada

## Abstract

We recently showed that streptozotocin (STZ) injections in rats lead to the development of painful peripheral diabetic neuropathy (PDN) accompanied by enhancement of Ca_V_3.2 T-type calcium currents (T-currents) and hyperexcitability in dorsal root ganglion (DRG) neurons. Here we used the classical peripherally acting T-channel blocker mibefradil to examine the role of Ca_V_3.2 T-channels as pharmacological targets for treatment of painful PDN. When administered intraperitoneally (i.p.), at clinically relevant doses, mibefradil effectively alleviated heat, cold and mechanical hypersensitivities in STZ-treated diabetic rats in a dose-dependent manner. We also found that Ca_V_3.2 antisense (AS)-treated diabetic rats exhibit a significant decrease in painful PDN compared with mismatch antisense (MIS)-treated diabetic rats. Co-treatment with mibefradil (9 mg/kg i.p.) resulted in reversal of heat, cold and mechanical hypersensitivity in MIS-treated but not in AS-treated diabetic rats, suggesting that mibefradil and Ca_V_3.2 AS share the same cellular target. Using patch-clamp recordings from acutely dissociated DRG neurons, we demonstrated that mibefradil similarly blocked T-currents in diabetic and healthy rats in a voltage-dependent manner by stabilizing inactive states of T-channels. We conclude that antihyperalgesic and antiallodynic effects of mibefradil in PDN are at least partly mediated by inhibition of Ca_V_3.2 channels in peripheral nociceptors. Hence, peripherally acting voltage-dependent T-channel blockers could be very useful in the treatment of painful symptoms of PDN.

## Introduction

Systemic administration of streptozotocin (STZ) has been shown to induce type 1 diabetes in rodents, resulting in peripheral diabetic neuropathy (PDN) often manifested as heat and mechanical hyperalgesia [1]. It is well known that Ca_V_3.2 T-type calcium channels (T-channels) contribute to the hyper-excitability of sensory neurons manifested as hyperalgesia and allodynia in rats with PDN [2], [3]. Blockade of Ca_v_3.2 T-type channels in STZ models of neuropathy, either by pharmacological means or by gene silencing using antisense technology, significantly reduces nociception in diabetic rats and mice *in vivo* [1]–[4]. Although several T-channel blockers exhibit effective antinociceptive activity in different animal models of neuropathic pain, due to the questionable selectivity of these agents, it remains unclear whether the observed effects are specific to T-channels or could be caused by the modulation of some off-targets. It is noteworthy that some new pharmacological agents, like 3, 5-dichloro-N-[1-(2, 2-dimethyl-tetrahydro-pyran-4-ylmethyl)-4-fluoro-piperidin-4-ylmethyl]-benzamide (TTA-P2) that are very selective T-channel blockers are effective analgesics in diabetic rats [5]. The studies with selective agents like TTA-P2 and related compounds are important to establish proof of concept for the use of T-channel blockers in the treatment of PDN. However, TTA-related drugs clearly exhibit sedation in animal models, likely due to the effects on T-channels in the central nervous system (CNS) [6]. This effect makes them unsuitable for long-term use in patients with chronic pain disorders [7]; thus there is need to examine the role of peripherally acting T-channel blockers in order to avoid side effects resulting from blockade of T-channels in the CNS. Hence, in our focus on T-channels in peripheral nociceptors as potential targets for PDN, we examined the role of the classical peripherally acting T-channel blocker mibefradil in combination with Ca_V_3.2-specific antisense oligonucleotides.

## Materials and Methods

### Chemicals and animals

All experimental protocols were approved by the University of Virginia Animal Care and Use Committee. All experiments were conducted in accordance with the *Guide for the Care and Use of Laboratory Animals* adopted by the U.S. National Institute of Health. Every effort was made to minimize animal suffering and the number of animals used. Using standard batteries of behavioral tests for mechanical and thermal hyperalgesia and allodynia, we established that retired breeder female and male rats exhibit similar pain sensitivity [8]. Thus, we used adult female Sprague–Dawley rats (retired breeders, 10–12 months old, weight 259±8 g) for the present study. Streptozotocin (STZ) was purchased from Sigma, St. Louis, MO. Previously published sequences [1], [9] of antisense Cav3.2 oligonucleotides (AS) and mismatched Cav3.2 oligonucleotides (MIS) were purchased from Eurofins MWG Operon. Morphine sulphate (administered i.p. at a dose of 10 mg/kg in sterile solution) was obtained from the University of Virginia pharmacy. Mibefradil (Sigma-Aldrich, St. Louis, MO), AS-Ca_V_3.2 and MIS-Ca_V_3.2 oligonucleotides where dissolved in appropriate sterile pH 7.4 buffer solution.

### Peripheral diabetic neuropathy model

To induce PDN, we intraperitoneally (i.p.) injected freshly dissolved STZ solution at pH 4.5 at a dose of 50 mg/kg, which causes severe hyperglycemia and pain-like behavior by the 4th week after injection [1]. Control rats received the same volume/kg i.p. of sterile saline (SAL). Heat and mechanical hypersensitivities and cold allodynia pharmacological and oligonucleotide testing were performed during the 4th week following STZ injections. All drug injections were performed in a blinded manner.

### Intrathecal Injections

We initiated intrathecal injections of either AS or MIS after we established stable heat and mechanical hyperalgesia concomitantly with elevated glucose blood levels (>600 mg/dl), at which point STZ-injected rats had developed PDN (day 21 post-STZ injection). Rats were maintained under isoflurane anesthesia (2% in oxygen delivered via nose cone) during the injection procedure. We injected into the L5–6 region of the spinal cord 12.5 µg/25 µl of either AS or MIS every 12 h for 4 consecutive days (total of 8 injections). All solutions were adjusted to pH 7.4 to avoid spinal cord irritation.

### Behavioral Assessment of Heat Nociception

The nociceptive response to heat stimulation was measured using a paw thermal stimulation system. Briefly, the system consists of a clear plastic chamber (10×20×24 cm) that sits on a clear elevated glass floor and is temperature regulated at 30°C using a paw thermal stimulation system [10]. Each animal is placed in the plastic chamber for 15 min to acclimate. A radiant heat source mounted on a movable holder beneath the glass floor is positioned to deliver a thermal stimulus to the plantar side of the hind paw. When the animal withdraws the paw, a photocell detects interruption of a light beam reflection and the automatic timer shuts off. This method has a precision of ±0.05 s for measurement of paw withdrawal latency in seconds (PWL). To prevent thermal injury, the light beam is automatically discontinued at 20 s if the rat fails to withdraw its paw. Pain testing was done before STZ or vehicle injection (normal baseline) and at the end of the third week after injection (peripheral neuropathy baseline) to establish development of PDN. The PWLs were noted after AS or MIS injections (marked as AS/MIS) to confirm the effect of knock-down treatment and just before the pharmacological studies with mibefradil or morphine (marked as 0 hr).

### Behavioral Assessment of mechanical sensitivity

To measure mechanical sensitivity, rats were placed in a clear plastic cage with a wire-mesh-bottom. The cage permits rats freedom of movement while allowing investigators access to their paws [11]. Von Frey filaments (Stoelting, Wood Dale, IL) were used to assess the mechanical threshold for paw withdrawal. These filaments are designated as the log10 (milligram weight required to cause bending ×10). We have found that applying the 5.18 filaments to the plantar surface of the foot causes a noxious response in female rats that results in an average of 4–5 paw withdrawal responses (PWRs) in 10 trials. Baseline withdrawal scores were determined in both paws to establish development of neuropathic pain prior to injections of AS, MIS, mibefradil or morphine using the same paradigm as for heat testing.

### Behavioral Assessment of cold allodynia

The procedure was described in detail in [12], [13]. Briefly, after a drop (50 µl) of acetone was sprayed onto the ventral side of the hind paw, we started to time the rats' reaction over the ensuing 20 s. No response (score = 0) was recorded if the rat did not withdraw, flick or stamp its paw. However, our observation period was increased to 40 s if the animal responded so that pain-related responses *per se* could be recorded. The scoring system was instituted following the criteria published by Flatters and Bennett [13]: 0, no response; 1, quick withdrawal, flick or stamp of the paw; 2, prolonged withdrawal or repeated flicking (at least two) of the paw; 3: repeated flicking of the paw with licking directed at the ventral side of the paw. The responses were obtained after acetone was applied alternately three times to each paw. Cumulative scores were generated with the minimum score being 0 (no response to any of the six trials) and the maximum possible score being 18 (repeated flicking and licking of paws on each of the six trials). Baseline cold scores were determined prior to and 3 weeks after injections of STZ. To assess the cooling effect of acetone we measured skin temperature post-acetone application and determined that there is about 3°C drop (from approximately 29.5°C to 26.5°C in SAL rats and from 28.0°C to 25.0°C in STZ rats over the course of 20 seconds; data not shown, n = 4 animals in each group). This is in accordance with previously published findings suggesting about a 4°C drop in skin temperature post-acetone application [14].

### Computerized analyses of the areas under the curve

To analyze the areas under the curves, we first plotted the combined mean values from right and left paws. Once the computerized graphs were created, we calculated the mean areas under the curve so that we could perform *vis-à-vis* comparisons of either heat or mechanical responses in SAL- and STZ- rats. The superimposed areas under the curve were calculated using ImageJ (NIH) software.

### Statistical analysis

Statistical analyses where performed using GraphPad Prism® software. We used two-way analysis of variance (ANOVA) to analyze within-subject variables, test session (before the administration of STZ or vehicle versus each post-treatment day) and between-subject variables (AS- or MIS-treated groups before and after administration of mibefradil or morphine). On the raw data, we used one-way ANOVA to confirm the efficacy of AS vs. MIS treatment. Relevant pair-wise comparisons were made and alpha levels were adjusted using the Bonferroni procedure when appropriate. We considered p<0.05 as being statistically significant. All data are presented as means ± SEM.

### Western Blot Analysis of Ca_V_3.2 channel protein expression

For the collection of lumbar DRGs, STZ-rats were deeply anesthetized with isoflurane and decapitated. Both right and left lumbar DRGs were rapidly extracted and dissected in phosphate buffer with a protease inhibitor cocktail (Roche, Germany), at which point they were rapidly frozen in liquid nitrogen. Tissue samples were homogenized in a phosphate buffer with protease inhibitor cocktail, sonicated and centrifuged at 12000 rpm for 10 min at 4°C to remove cell debris. Supernatants were collected and protein concentrations were determined using the Lowry method. Samples were combined with 2× Laemmli buffer (Sigma-Aldrich, St. Louis, MO, USA), boiled for 5 minutes, loaded into a 7.5% polyacrylamide gel and electrophoresed. Separated proteins were transferred to nitrocellulose membranes and blocked with 5% non-fat milk at room temperature for 1 hour. The membranes were incubated overnight in primary goat polyclonal antibodies directed at the C-terminus of the Ca_V_3.2 channel (sc-16263, Santa Cruz Biothechology Inc, CA) or at the house-keeping protein actin (Sigma-Aldrich) at respective dilutions of 1∶100 and 1∶11,000, respectively. Appropriate horseradish peroxidase–conjugated secondary antibodies (anti-rabbit IgG, 1∶10,000 for actin and 1∶5000 for the Ca_V_3.2 channel; Santa Cruz Biotechnology Inc, CA) were applied for 1 hour. The membranes were developed using enhanced chemiluminescence (ECL) detection reagents (GE Healthcare Life Sciences, Piscataway, NJ) and band density was quantified using Syngene Gel documentation, G-box analysis software (Syngene, USA). Densities for Ca_V_3.2 channel bands were normalized to bands for actin, which was used as a housekeeping protein. Since saline and MIS treatments caused no changes in the expression of Ca_V_3.2 channel protein expression, the data were combined and are presented as the MIS-control group. To determine the specificity of anti-Ca_V_3.2 antibody, we performed Western Blot experiments under identical conditions with human embryonic kidney (HEK-293) cells stably transfected with either human Ca_V_3.1 or Ca_V_3.2 isoforms of T-channels. A main band of strong immunoreactivity at approximately 250 KD in the membrane fraction was present only in Ca_V_3.2-transfected HEK-293 cells, not Ca_V_3.1-transfected HEK-293 cells (n = 4 experiments in each group, data not shown).

### Electrophysiological recordings

DRG cells from adolescent rats were prepared as previously described [15]. For recording, cells were plated onto uncoated glass coverslips, placed in a culture dish, and perfused with external solution. All *in vitro* experiments were done at room temperature.

Recording electrodes were pulled from borosilicate glass microcapillary tubes (Drummond Scientific, Broomall, PA); when filled with solution, they had resistances between 1 and 4 MΩ. We made recordings using an Axopatch 200B patch-clamp amplifier (Molecular Devices, Foster City, CA). Digitization of membrane voltages and currents was controlled using a Digidata 1322A interfaced with Clampex 8.2 or 9.0 (Molecular Devices). We analyzed data using Clampfit 8.2 or 9.0 (Molecular Devices) and Origin 7.0 (Microcal Software, Northampton, MA). Currents were low pass-filtered at 2–5 kHz. We took series resistance and capacitance values directly from readings of the amplifier after electronic subtraction of the capacitive transients. Series resistance was compensated to the maximum extent possible (usually 50%–80%). Multiple independently controlled glass syringes served as reservoirs for a gravity-driven perfusion system.

The external solution contained (in mM) 152 TEA-Cl, 2 CaCl_2_, and 10 HEPES, adjusted to pH 7.4 with TEA-OH. For studies of well-isolated and well-clamped T-currents in acutely isolated DRG cells, we used only fluoride (F^−^)-based internal solution to facilitate high voltage-activated (HVA) Ca^2+^ current rundown; this internal solution contained (in mM) 135 TMA-OH, 40 HEPES, 10 EGTA, and 2 MgCl_2_, adjusted to pH 7.2 with hydrogen fluoride (HF). The amplitude of the T-current at any given potential was measured from the end of the pulse to its peak. Mibefradil was prepared as a 100 mM stock solution and freshly diluted to the final concentrations in the external solution at the time of experiments.

The percent reductions in peak T-current at various concentrations of mibefradil were used to generate concentration-response curves. Mean values were fit to the following Hill-Langmuir function:
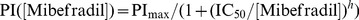
(1)where PI_max_ is the maximal percent inhibition of peak current by mibefradil, IC_50_ is the concentration that produces 50% inhibition, and *h* is the apparent Hill-Langmuir coefficient for inhibition. The fitted values are reported with >95% linear confidence limits.

To study the effects of mibefradil on steady-state inactivation of T-channels, currents are evoked every 7 seconds by test steps to −40 mV after 3.5-sec pre-pulses to potentials ranging from −110 mV to −45 mV in 5 mV increments.

The voltage dependence of steady-state inactivation was described with a single Boltzmann distribution of the following form:
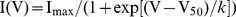
(2)where I_max_ is the maximal activatable current, V_50_ is the voltage at which half the current is inactivated, and *k* is the voltage dependence (slope) of the distribution.

## Results

### Metabolic parameters

Body weights were assessed daily after SZT- or saline (SAL) injection. While body weights of control animals injected i.p. with saline (pH = 7.4) did not fluctuate much, there was about 10–18% weight loss in STZ-injected animals (data not shown).

**Figure 1:**
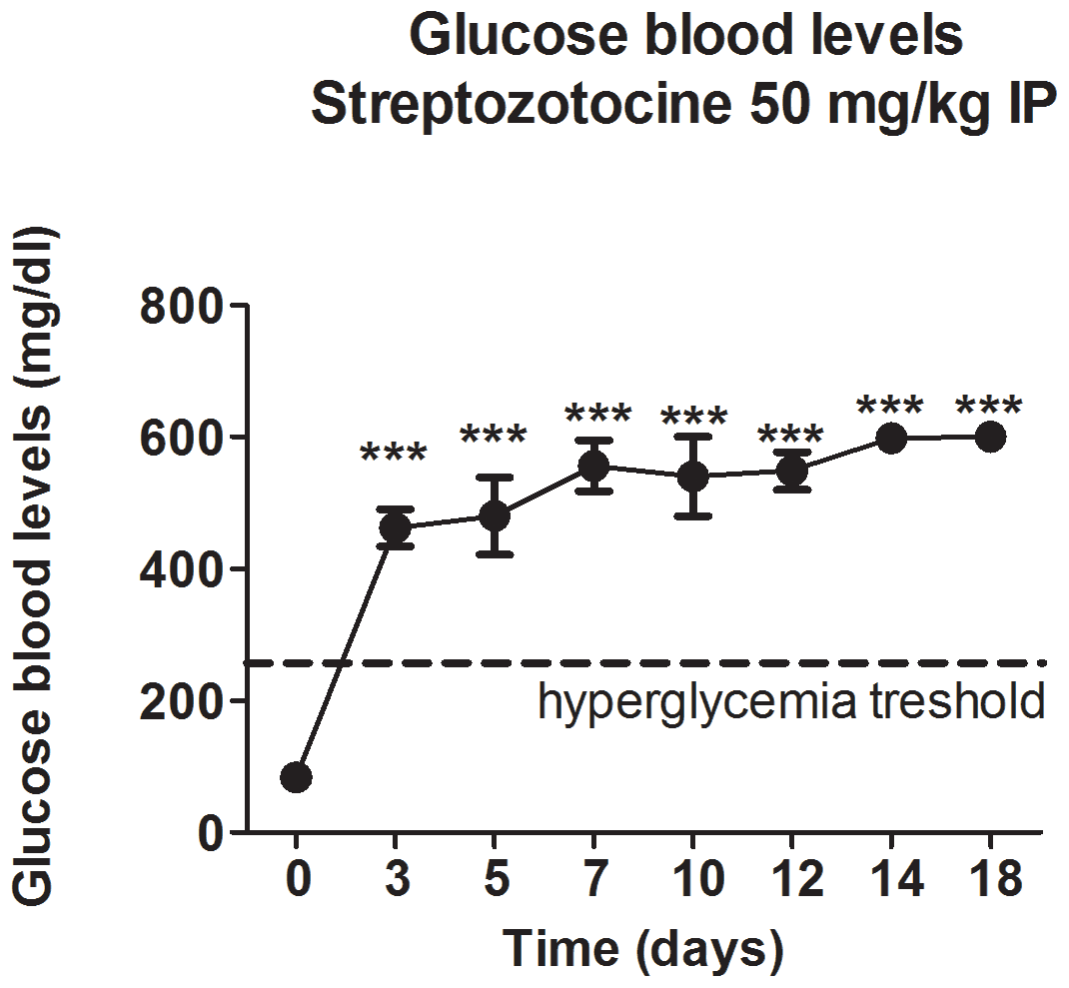
Systemically administered streptozotocine (STZ) induces severe hyperglycemia in adult female rats. Intraperitoneally (IP) injected STZ, at 50 mg/kg, resulted in high blood glucose levels within the first three days. The blood glucose levels reached 600 mg/dl (the limits of the glucometer) within two weeks post-treatment and remained elevated thereafter (***, p<0.001 compared to the baseline glucose level; dashed line marks the hyperglycemia threshold in rats; n = 5 rats per data point).

Before assigning rats to either STZ (experimental) or SAL (control) groups, we determined their baseline blood glucose to be about 80 mg/dl. While blood glucose levels in the SAL group remained around that level throughout the experimental period (data not shown), STZ-animals developed severe hyperglycemia (over 400 mg/dl) within the first three days and it continued to worsen, exceeding 600 mg/dl (the limitation of the glucometer) in about two weeks (Figure 1). In addition, STZ-animals had increased water and food consumption during the first three weeks as well as an increase in urine output (thus mimicking clinical signs of diabetes, e.g. polydipsia, polyphagia and polyuria), but without any signs of general sickness due to ketoacidosis. Hence, we chose to conduct our behavioral tests during the 4^th^ week (21 days post-STZ injection) when the clinical picture of poorly controlled diabetes was confirmed and yet general well-being of the animals was acceptable so as not to confound the studies of neuropathic pain behavior.

### STZ-induced painful diabetic neuropathy is manifested by heat and mechanical hyperalgesia that is alleviated with mibefradil and Ca_V_3.2 antisense

**Figure 2:**
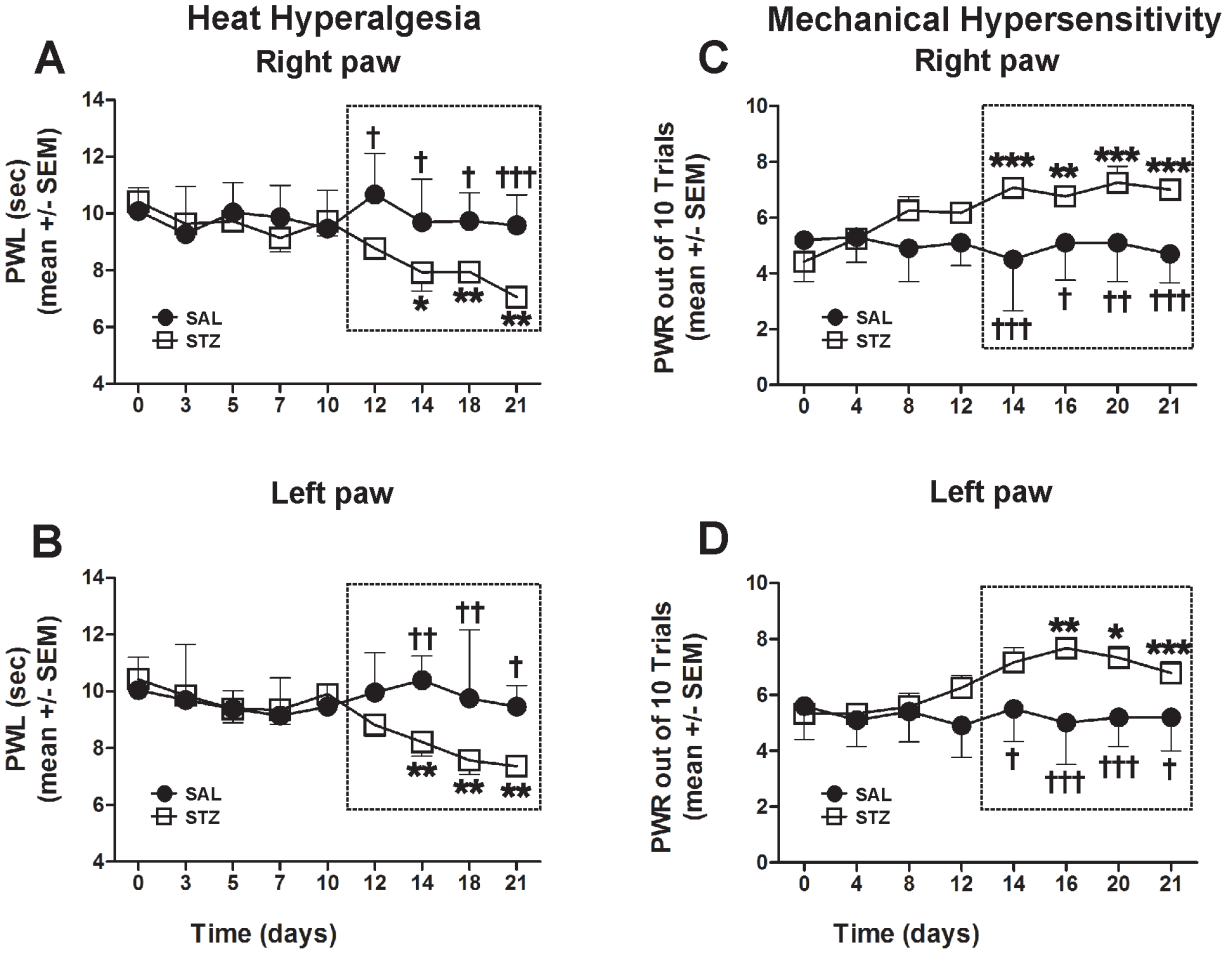
Diabetic rats develop significant heat hyperalgesia (A, C) and mechanical hypersensitivity (B, D). PWLs and PWRs were recorded starting with assessment of the baseline before STZ administration (0 day) followed by periodical assessment for the next 21 days (A, Right paw, B, Left paw). Although a decline in PWL was noted first on day 12, it did not reach significance until day 14 when it approached a steady state (marked with dashed rectangle) of heat hyperalgesia as compared with the baseline recordings (*, p<0.05; **, p<0.01). The PWLs recorded in SAL animals revealed no change compared with the baseline but a significant change compared with those of STZ animals from day 12 (A) and day 14 (B) (†, p<0.05; ††, p<0.01; †††, p<0.001). Note similar latencies recorded in left and right paws indicating the systemic nature of the disease (A and B). The steady rise in PWRs indicative of mechanical hypersensitivity started on day 12; PWRs became significant from 14 days post-STZ treatment as compared with the baseline PWR recordings (*, p<0.05; **, p<0.01; ***, p<0.001). The PWRs recorded in SAL animals revealed no changes compared with the baseline but a significant change compared with STZ animals from day 14 (†, p<0.05; ††, p<0.01; †††, p<0.001; n = 5 STZ rats and n = 5 SAL rats per data point in A and B; n = 6 STZ rats and n = 5 SAL rats per data point in C and D).

To study the development of PDN *in vivo*, we examined heat (PWLs; Figure 2A and B) and mechanical (PWRs; Figure 2C and D) sensitivities in STZ- and SAL-rats. Baseline heat and mechanical sensitivities were assessed before the assigned treatment (marked as day 0). Note similar responses recorded in the right (A and C) and left paws (B and D). As shown in Fig. 2A and B, PWLs started to decline approximately two weeks post STZ-injection (by about 20–25% compared with the baseline, *, p<0.05; **, p<0.01) and remained significantly decreased throughout the testing period, suggesting the development of heat hyperalgesia. Note that significant heat hyperalgesia (outlined with dashed rectangle) occurs about a week after the development of severe hyperglycemia (as shown in Fig. 1). The PWLs recorded from the SAL-rats during the same time period reveled minimal changes compared with the baseline and consequently, significant changes compared with PWLs in STZ-rats (†, p<0.05; ††, p<0.01; †††, p<0.001 starting from day 12- right paw and day 14- left paw).

Likewise, PWRs started to increase soon after STZ treatment and became significantly elevated in about 2 weeks (around 40–60% increase compared with the baseline PWRs; *, p<0.05; **, p<0.01; ***, p<0.001), suggesting the development of significant mechanical hypersensitivity (dashed rectangle; Fig. 2C and D). The PWRs recorded from the SAL-rats during the same time period reveled minimal changes compared with the baseline and consequently, significant changes compared with PWRs in STZ-rats (†, p<0.05; ††, p<0.01; †††, p<0.001 starting from day 14).

We conclude that the onsets of heat hyperalgesia and mechanical hypersensitivity, the hallmarks of PDN, coincide with the development of severe hyperglycemia (Fig. 1) although (as expected) the development of the behavioral indices of PDN lagged behind the development of metabolic indices of diabetes.

**Figure 3:**
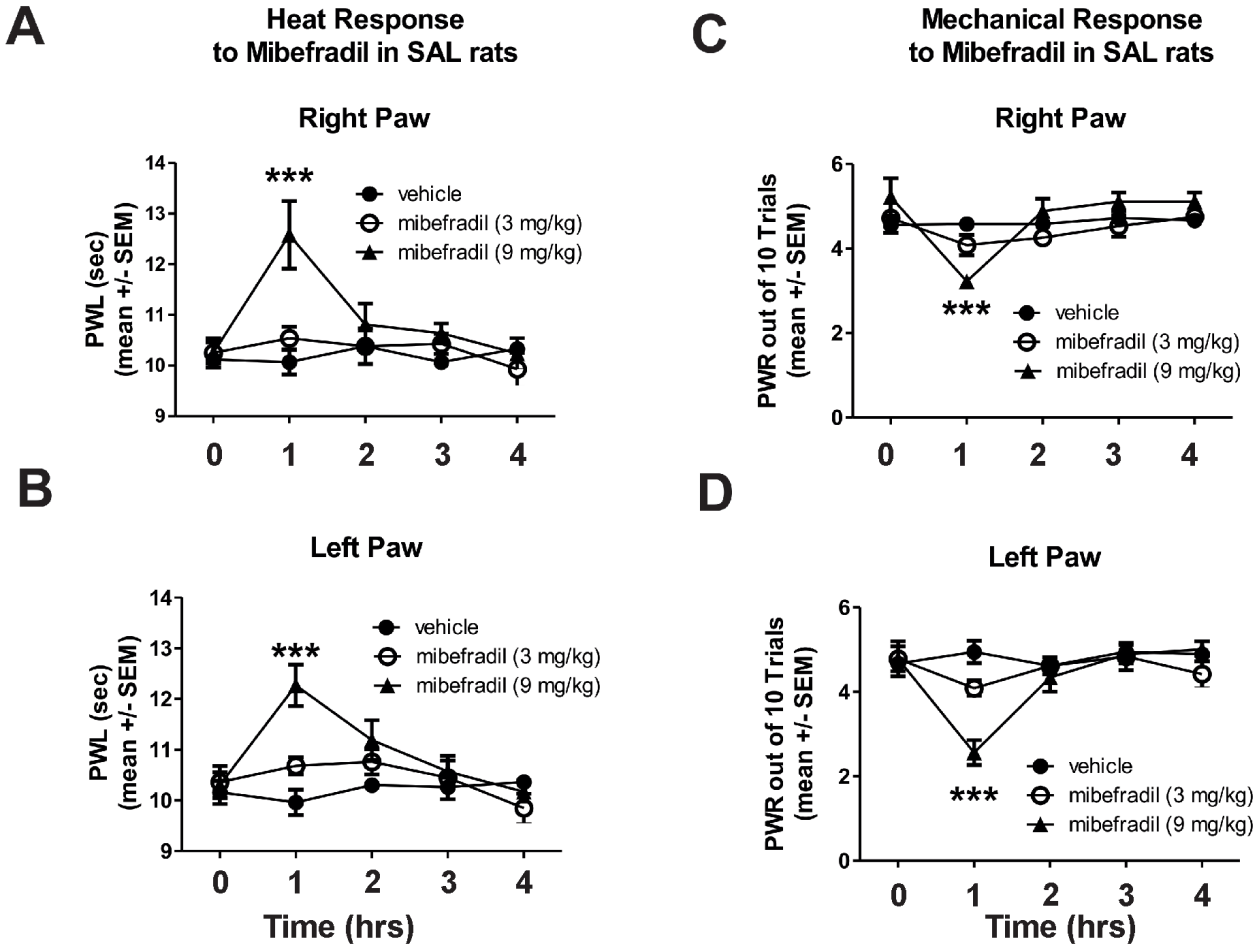
Mibefradil induces transient alleviation of heat and mechanical sensitivity in non-diabetic rats. Mibefradil at 9/kg but not at 3 mg/kg causes significant, although transient, decrease in heat (***, p<0.001 at 1 hr compared to vehicle in both left and right paws; Panel A, right paw; B, left paw) and mechanical sensitivity (***, p<0.001 at 1 hr compared to vehicle in both paws; Panel C, right paw; D, left paw) in control (SAL)-rats (n = 3–9 rats per data point).

To investigate the role of peripheral T-channels in the development of painful PDN, we used two clinically relevant doses, 3 and 9 mg/kg, of mibefradil injected intraperitoneally (i.p.). At these two doses, mibefradil was shown previously to have transient analgesic effects in healthy rats without affecting their sensorimotor ability [16]. Furthermore, it has been shown that at these doses, mibefradil exhibits beneficial cardiovascular protective effects in rats [17]–[19]. Here we show that mibefradil at 9 mg/kg but not 3 mg/kg causes a significant, although transient, decrease in heat (***, p<0.001 at 1 hr compared with vehicle in both left and right paws; Fig. 3A, right paw; 3B-left paw) and mechanical (***, p<0.001 at 1 hr compared with vehicle in both paws; Fig. 3C, right paw; 3D, left paw) sensitivity in control (SAL)-rats.

**Figure 4:**
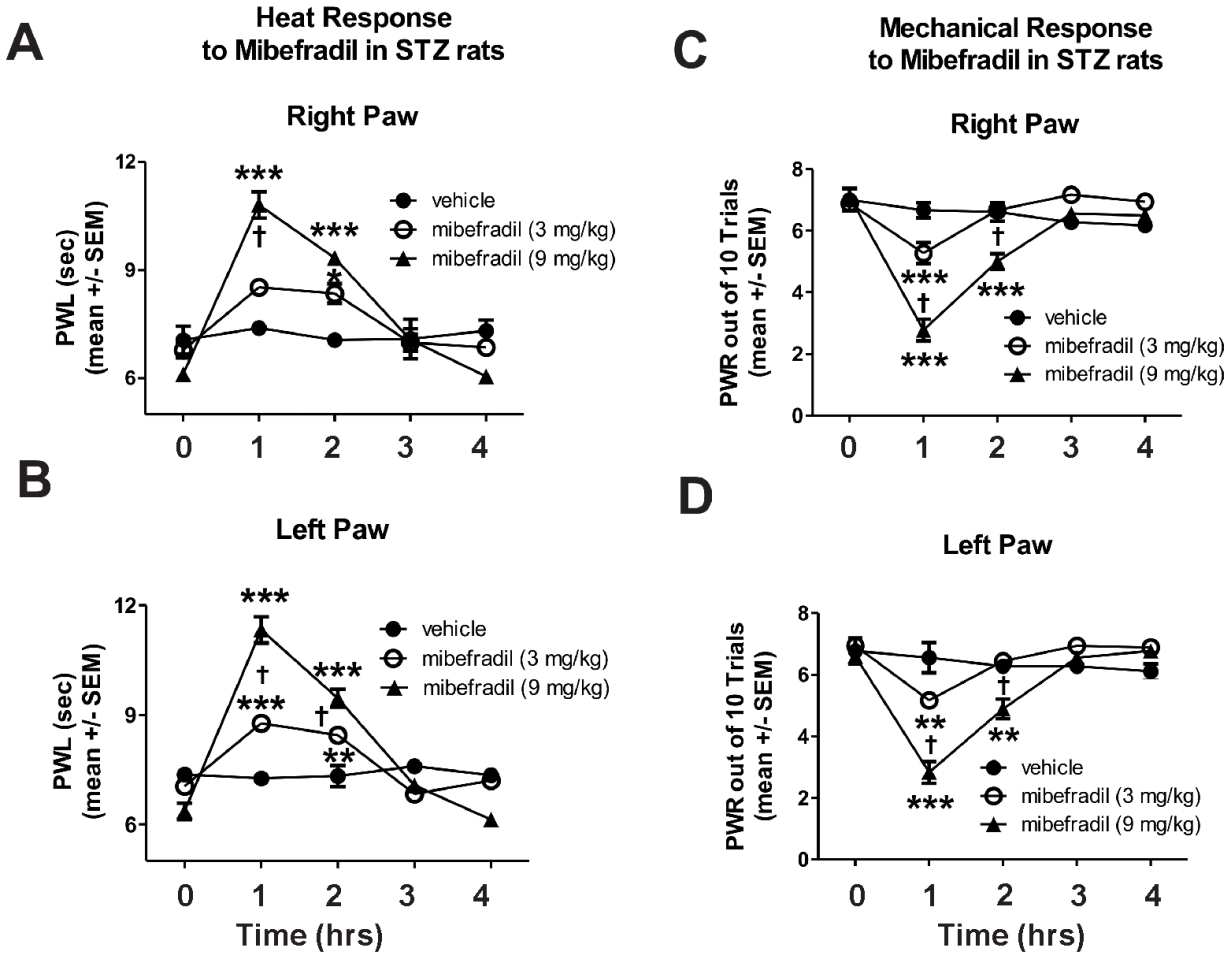
Mibefradil induces longer-lasting alleviation of heat hyperalgesia and mechanical hypersensitivity in diabetic rats. Mibefradil treatment was performed on day 21 when PDN was confirmed (see Figure 2). Mibefradil causes a dose-dependent profound effect on heat (**A**, right paw; **B**, left paw) and mechanical (**C**, right paw; **D**, left paw) hypersensitivity. The lower dose of mibefradil (3 mg/kg) caused a significant alleviation of heat hyperalgesia for the first couple of hours compared with a vehicle treatment (*, p<0.05; **, p<0.01; *** p<0.001). A higher dose (9 mg/kg) of mibefradil induced an even more profound effect (***, p<0.001 compared with vehicle); (†, p<0.05, ††† p<0.001, in mibefradil at 3 mg/kg compared to mibefradil at 9 mg/kg). Similarly, mibefradil induced dose-dependent, significant (both at lower and higher doses) alleviation of mechanical hypersensitivity in diabetic rats that lasted a couple of hours (**, p<0.01 and ***, p<0.001 compared with vehicle; †††, p<0.001 mibefradil at 3 mg/kg compared with mibefradil at 9 mg/kg) (n = 3–6 rats per data point).

However, in diabetic STZ-treated rats (Figure 4) with confirmed PDN (note the 0 h time point PWLs and PWRs compared to 0 h time point in Figure 3), mibefradil had a dose-dependent and more profound effect on heat and mechanical hypersensitivity. For example, the lower dose of mibefradil (3 mg/kg) that was ineffective in SAL-rats caused significant alleviation of heat hyperalgesia in STZ-rats for the first couple of hours compared with a vehicle treatment (*, p<0.05, **, p<0.01 and ***, p<0.001; Fig. 4-A, right paw; 4-B, left paw) whereas at a higher dose (9 mg/kg), mibefradil induced even more profound and longer lasting effect (***, p<0.001 compared with vehicle in both paws; †, p<0.05 and †††, p<0.001, mibefradil at 3 mg/kg compared with mibefradil at 9 mg/kg). Note that PWLs and PWRs recordings at 0 h time point are as shown in Fig. 2 on day 21 post-STZ injection.

Similarly, mibefradil induced significant and dose-dependent alleviation of mechanical hypersensitivity in diabetic rats that was more pronounced and longer lasting than that observed in SAL-rats (Fig. 4- C, right paw; 4-D, left paw; **, p<0.01 and ***, p<0.001 compared with vehicle; †††, p<0.001 mibefradil at 3 mg/kg compared with mibefradil at 9 mg/kg).

**Figure 5:**
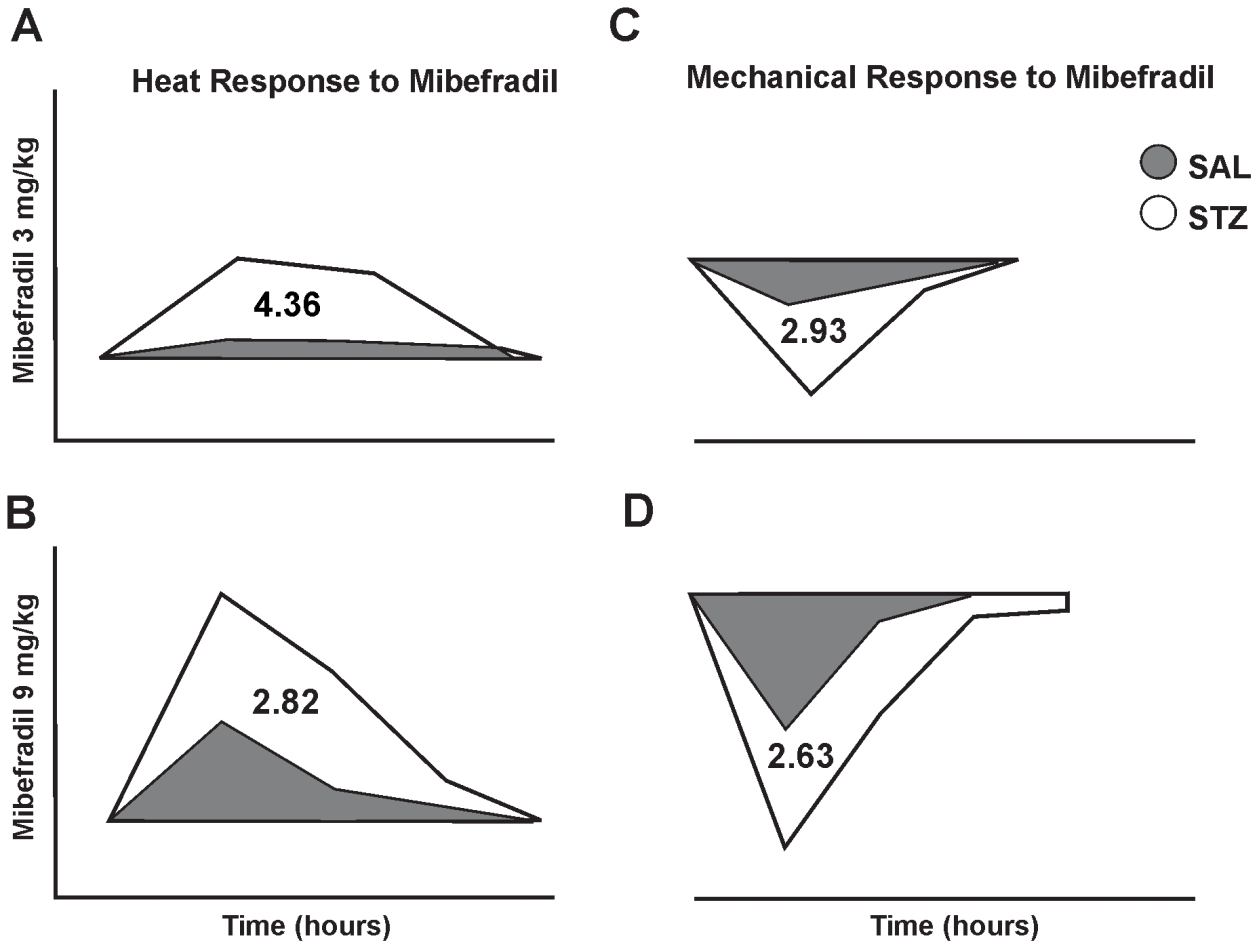
Schematic representation of the areas under the curves calculated from the thermal and mechanical responses to mibefradil recorded from SAL- and STZ-rats. Using computerized analyses of the actual data presented in Figs. 3 and 4, we calculated the average area under the curve for right and left paws in SAL- (shaded area) and STZ-rats (open area) in order to quantify and compare the antinociceptive effectiveness of mibefradil in SAL- and STZ-rats. At lower dose (3 mg/kg) we report higher effectiveness of mibefradil in alleviating heat (4.36-fold) (A) and mechanical sensitivity (2.93-fold) (C) in STZ-rats compared with SAL-rats. At a higher dose (9 mg/kg), we also report higher effectiveness of mibefradil [2.82-fold for heat sensitivity (B) and 2.63-fold for mechanical sensitivity (D)], although the effect is somewhat less impressive than that with the lower dose, most likely due to a ceiling effect.

These findings suggest that mibefradil is more effective in alleviating thermal and mechanical sensitivity in STZ- compared with SAL-rats in terms of duration and dose required to achieve the desired effect. To confirm this notion we conducted computerized analyses of the average areas under the curve for both paws and compared the thermal and mechanical response in SAL- and STZ-rats to either 3 or 9 mg/kg of mibefradil. As shown in schematic representation (Fig. 5), we found that a lower dose of mibefradil was about 3- to 4-fold more effective in STZ- compared with SAL-rats, both in terms of alleviating heat (A) and mechanical hyperalgesia (C). Interestingly, at a higher dose, the effectiveness of mibefradil (B and D) was somewhat less impressive (slightly less than 3-fold difference between SAL- and STZ-rats), presumably due to a ceiling effect.

**Figure 6:**
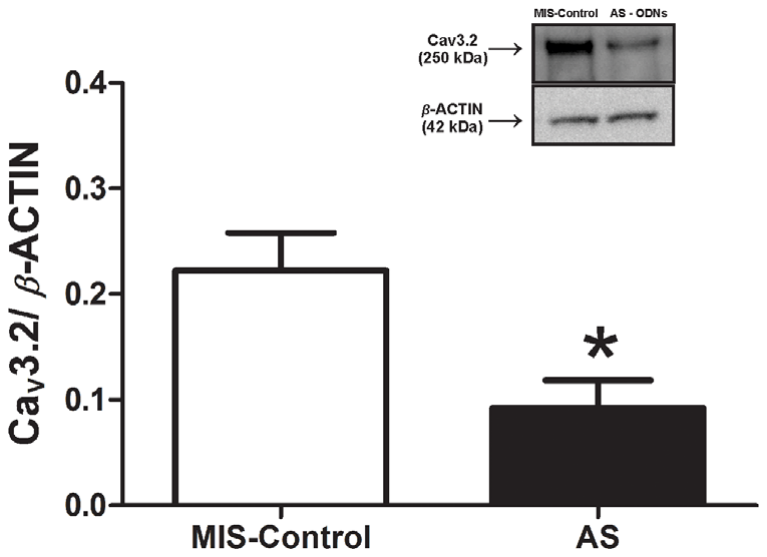
Intrathecal lumbar administration of AS induces significant down-regulation of the Ca_V_3.2 channel protein expression in lumbar DRGs of STZ rats. Protein expression of Ca_V_3.2 channel was analyzed and expressed as a ratio to that of the housekeeping protein β-actin. The bar graph shows that in AS-Ca_V_3.2-treated rats (closed bar) we detected, on average, about a 60% decrease in Ca_V_3.2 channel expression as compared with that of the MIS-control group (open bar; *, p<0.05). The protein measurement was performed 10 hours after the last AS-Ca_V_3.2 injection. (n = 5 rats in MIS-control group; n = 4 rats in AS group). The insert illustrates original immunoblots from the representative experiments. (Since there was no difference in protein expression of Ca_V_3.2 channel in STZ rats treated with either MIS or saline, the findings are compiled and presented as MIS-Control).

**Figure 7:**
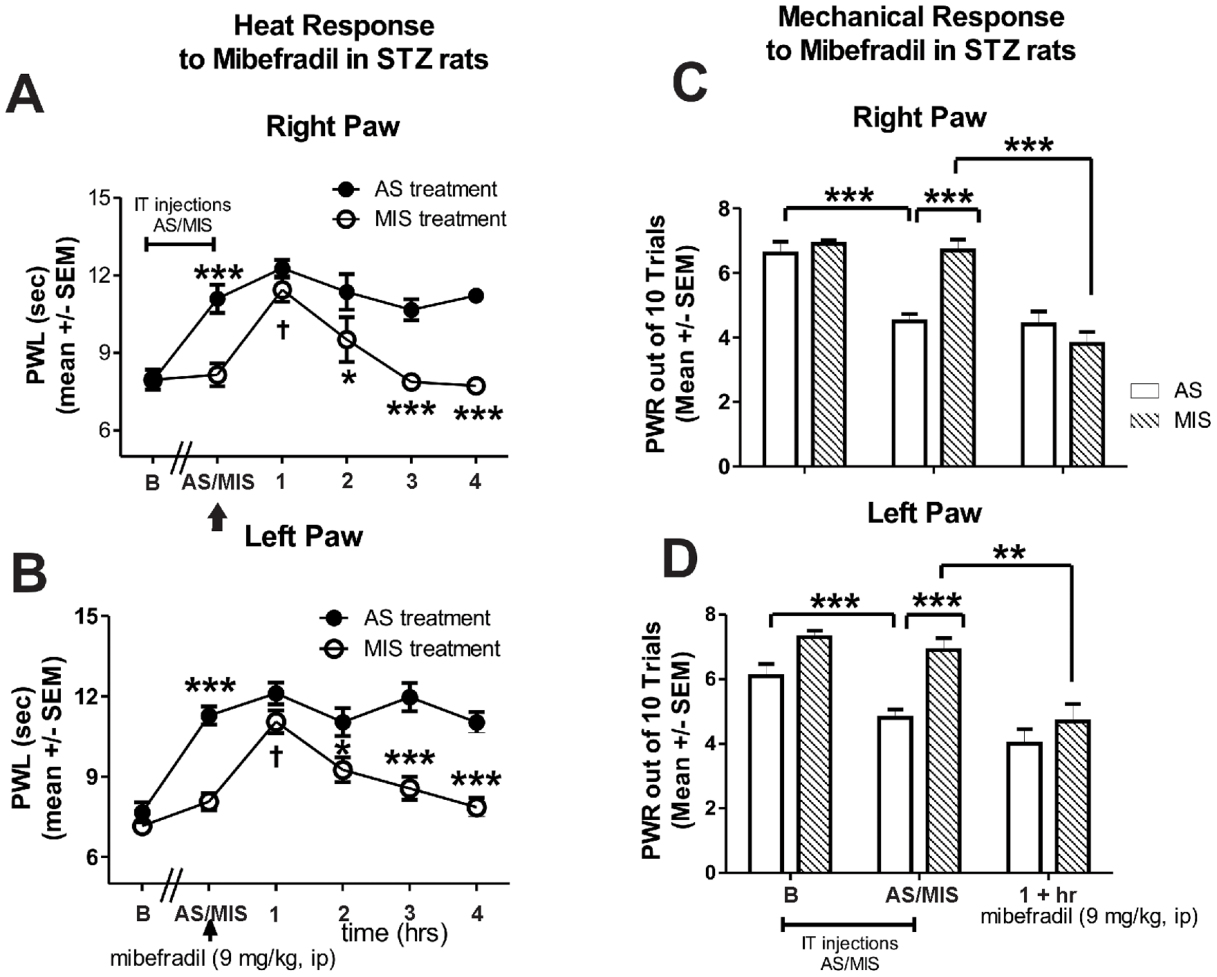
Knock-down of Ca_V_3.2 abolishes mibefradil-induced alleviation of heat hyperalgesia and mechanical hypersensitivity in diabetic rats. Ca_v_3.2 T-channels in sensory neurons were knocked-down with intrathecal administration (every 12 hrs for a total of 4 doses) of oligodeoxynucleotide (AS) specific for the Ca_v_3.2 T-channels. Controls were given mismatch oligodeoxynucleotide (MIS). Heat hyperalgesia was quantified with PWLs (A, right paw; B, left paw) and mechanical hypersensitivity was measured using PWRs (C, right paw; D, left paw). Compared with baseline recordings (marked as B), AS-treated diabetic rats exhibited a significant increase in PWLs (***, p<0.001) and a significant decrease in PWRs (***, p<0.001; marked as AS/MIS). At that point, mibefradil was administered (indicated with an arrow) and heat hyperalgesia was assessed hourly. Mibefradil (at 9 mg/kg i.p.) had no further effect to alleviate heat hyperalgesia; i.e., PWLs recordings remained stable (and significantly elevated compared with the MIS-treated STZ rats; *, p<0.05; ***, p<0.001) throughout the four-hour period post-mibefradil administration (closed circles). In MIS-treated diabetic rats, mibefradil induced a transient increase in PWLs (†, p<0.05 at 1 hr post-injection). Mechanical sensitivity assessment was initiated 1 h post mibefradil treatment when the effect was shown to be most significant (as indicated in Fig. 3C and D). PWRs in AS-treated diabetic rats post-mibefradil injection remain unchanged whereas in MIS-treated diabetic rats there was a significant effect of mibefradil marked by a decrease in PWRs (***, p<0.001, right paw; **, p<0.01, left paw; n = 5 rats per data point).

To further validate the Ca_V_3.2 T-channel as an important cellular target for pain-alleviating effects of mibefradil in PDN, we administered intrathecally every 12 hrs for a total of 4 doses an antisense oligodeoxynucleotide (AS) specific for the Ca_v_3.2 T-channel or a mismatch oligodeoxynucleotide (MIS) as a control. At the end of the antisense/mismatch treatment we established two important elements: first, that the AS application resulted in a significant down-regulation of Ca_V_3.2 T-channel protein expression in lumbar DRGs compared with MIS-Control (Fig. 6, *, p<0.05, n = 5 rats in MIS-control group and 4 rats in AS group), and second, that the AS-treated diabetic rats exhibited a significant decrease in heat and mechanical hypersensitivity as compared with MIS-treated diabetic rats (Fig. 7; 0 h time point marked as AS/MIS). Once the hypersensitivity was confirmed (**, p<0.01; ***, p<0.001 AS vs. MIS in both paws), mibefradil was administered (indicated with an arrow) and pain phenotype was assessed on an hourly basis. As illustrated in Fig. 7 (A, right paw; B, left paw) co-treatment with mibefradil (9 mg/kg i.p.) in AS-treated STZ-rats had no further effect on heat sensitivity, i.e., PWLs' recordings remained stable throughout the four-hour period post-mibefradil administration, suggesting that mibefradil and AS likely share the same cellular target. On the other hand, MIS-treated STZ-rats responded to mibefradil similarly to what we observed in STZ-rats that did not receive either AS or MIS treatment (as shown in Fig. 4 A-right paw; B-left paw), i.e., mibefradil induced a significant increase in PWLs (†††, p<0.001 at 1 hr post-mibefradil injection in MIS STZ-rats compared to 0 h), further suggesting that the Ca_V_3.2 T-channel is mibefradil's cellular target for alleviating thermal hypersensitivity in PDN (*, p<0.05 and ***, p<0.001 in AS vs. MIS rats post-mibefradil injection). Similarly, when mechanical sensitivity was assessed (Fig. 7; C, right paw; D, left paw), we observed significant alleviation of mechanical hypersensitivity in AS-treated diabetic rats (**, p<0.01; ***, p<0.001 in AS vs. MIS rats) and the effect was unchanged with mibefradil treatment. However, in MIS-treated diabetic rats in which mechanical hypersensitivity was unchanged compared with the baseline, mibefradil caused a significant decrease in PWRs (***, p<0.001, right paw; **, p<0.01, left paw), suggesting that Ca_V_3.2 T-channels play an important role in mechanical hypersensitivity and are a likely cellular target of mibefradil's anti-PDN effects. Note that the assessment of mechanical sensitivity was initiated 1 h post mibefradil treatment when the effect was shown to be most significant (Fig. 3C and D).

Although not included in Fig. 7, our previously published work with SAL-AS-rats confirmed the importance of Ca_V_3.2 T-channels in the development of thermal and mechanical hyperalgesia while showing a complete lack of pain phenotype in SAL-MIS-rats [1].

**Figure 8:**
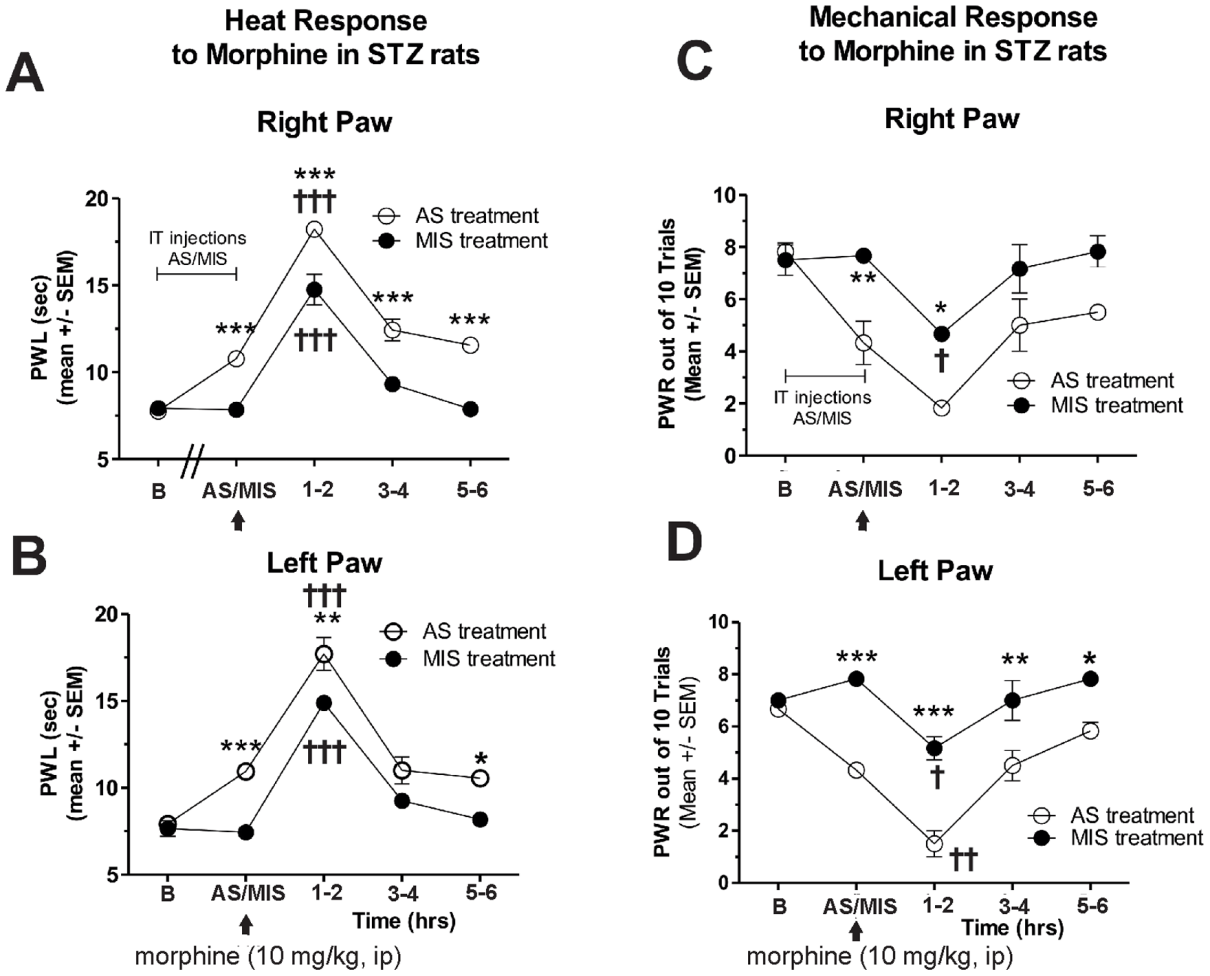
Knock-down of Cav3.2 has no effect on morphine-induced alleviation of heat and mechanical hypersensitivity. Ca_v_3.2 T-channels in sensory neurons were knocked down with intrathecal administration (every 12 hrs for a total of 4 doses) of oligodeoxynucleotide (AS) specific for the Ca_v_3.2 T-channels. Controls were given mismatch oligodeoxynucleotide (MIS). Heat hyperalgesia was quantified with PWLs (A, right paw; B, left paw) and mechanical hypersensitivity was measured using PWRs (C, right paw; D, left paw). The response to morphine (10 mg/kg, i.p.) in AS-treated diabetic rats mimicked the response in MIS-treated diabetic rats in that both groups exhibited change of PWLs and PWRs at around 1–2 hrs post morphine injection (†, p<0.001 compared with before injection in A and B; †, p<0.05 compared to before injection in C and D) except that there was a notable upward shift in the PWLs and a downward shift in the PWRs in AS-treated diabetic rats due to Ca_V_3.2 T-channel knock down (*, p<0.05; **, p<0.01; ***, p<0.001 in AS vs. MIS treatment; n = 3 rats per data point).

To probe further the mechanism of PDN in AS- or MIS-treated diabetic animals, we examined the antinociceptive response to a prototype opioid agent, morphine (10 mg/kg, i.p.). We found that morphine provided a similar decrease in heat nociceptive responses (Fig. 8A, right paw; 8B, left paw; †††, p<0.001 at 1–2 h after morphine injection) and mechanical nociceptive responses (Fig. 8C, right paw, †, p<0.05; and 8D, left paw, †, p<0.05; ††, p<0.01) in both AS- and MIS-treated diabetic groups, indicating that morphine's anti-nociceptive effects most likely are not mediated through Ca_v_3.2 T-channels. Note the significant upward shift in PWLs (A and B) and a significant downward shift in PWRs (C and D) in AS- compared with MIS-treated diabetic rats, indicating the shift in pain baseline due to Ca_V_3.2 T-channel knock-down (*, p<0.05; **, p<0.01; ***, p<0.001).

### STZ-induced painful diabetic neuropathy is manifested by cold allodynia that is alleviated by mibefradil and Ca_V_3.2 antisense but not by morphine

**Figure 9:**
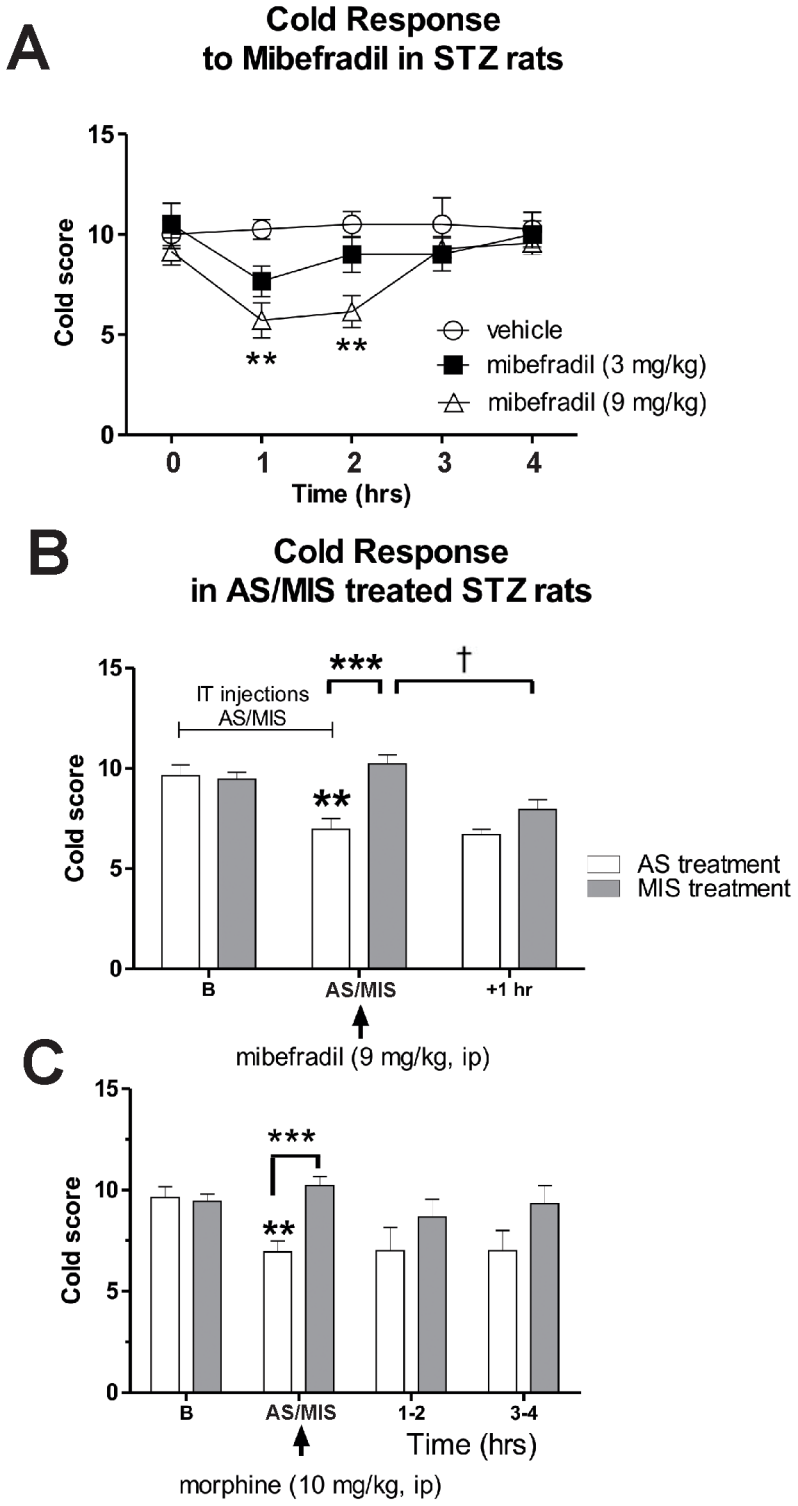
Mibefradil and Cav3.2 antisense are effective in alleviating diabetic cold allodynia. Ca_v_3.2 T-channels in sensory neurons were knocked down with intrathecal administration (every 12 hrs for a total of 4 doses) of oligodeoxynucleotide (AS) specific for the Ca_v_3.2 T-channels. Controls were given mismatch oligodeoxynucleotide (MIS). Cold allodynia was quantified using cold score (see Methods). **A**) Mibefradil induced dose-dependent alleviation of cold allodynia in diabetic rats compared with vehicle treatment. At 9 mg/kg, i.p., it caused approximately a 45% decrease in pain scores (**, p<0.01) compared with vehicle treatment, an effect that lasted a couple of hours. Although mibefradil, at 3 mg/kg, i.p., induced a slight decrease in cold scores, the effect was very transient and non-significant. **B**) AS-Ca_V_3.2 treatment had significant antiallodynic effect compared with the baseline (†††, p<0.001) and MIS-treatment (***, p<0.001). The antiallodynic effect of mibefradil was blocked with AS-Ca_V_3.2 (i.e., cold scores in AS-treated animals before and after mibefradil injection were not different), but not with MIS-Ca_V_3.2 (i.e. there was a significant decrease in cold scores in MIS-treated animals after mibefradil treatment compared with cold scores before the treatment; †, p<0.01). **C**) Unlike mibefradil, morphine was completely ineffective in alleviating cold allodynia in both AS- and MIS-diabetic rats at any of the time points. (††, p<0.01 in AS-treated diabetic animals compared with the baseline; ***, p<0.001 compared with MIS-treated diabetic animals) (n = 3–11 rats per data point).

Since cold allodynia is another important symptom of PDN [20], [21] and the importance of T-channels in its development remains unknown, we explored the putative roles of T-channels in cold allodynia in STZ-treated rats. Unlike SAL-treated rats that do not have any response to cold stimulus, STZ-rats when tested on the 21^st^ post-STZ treatment day, show obvious signs of cold allodynia [baseline (B) cold scores around 10 noted at 0 hrs; Fig. 9]. Once cold allodynia was confirmed, we performed AS/MIS and pharmacological experiments with mibefradil or morphine during the 4^th^ week (starting from the 21^st^ day post-STZ injection) as described for heat and mechanical testing. We found that mibefradil induced dose-dependent alleviation of cold allodynia in diabetic rats as compared with vehicle treatment. For instance, mibefradil, at 9 mg/kg i.p., caused an approximate 45% decrease in pain scores (**, p<0.01) compared with vehicle treatment, an effect that lasted a couple of hours (Fig. 9A). Similar to the effects of mibefradil on heat and mechanical hypersensitivity, the antiallodynic effect of mibefradil was not detected in AS-treated STZ-rats, but was significant in MIS-treated STZ rats (†, p<0.05 before mibefradil vs. 1+ post-treatment; Fig. 9B), again suggesting that AS and mibefradil share the same cellular target for alleviation of cold allodynia. Note a significant decrease in cold allodynia in AS-treated STZ rats when compared to baseline recordings (†††, p<0.001) and compared with MIS-treated STZ-rats (***, p<0.001). Finally, we show that unlike mibefradil, morphine was completely ineffective in alleviating cold allodynia in both AS- and MIS-diabetic rats (Fig. 9C). Note that there was a similar decrease in cold allodynia in AS-treated STZ rats compared to baseline recordings (††, p<0.01) and compared to MIS-treated STZ-rats (***, p<0.001).

### Mibefradil inhibits isolated DRG T-currents in diabetic rats in a voltage-dependent manner

**Figure 10:**
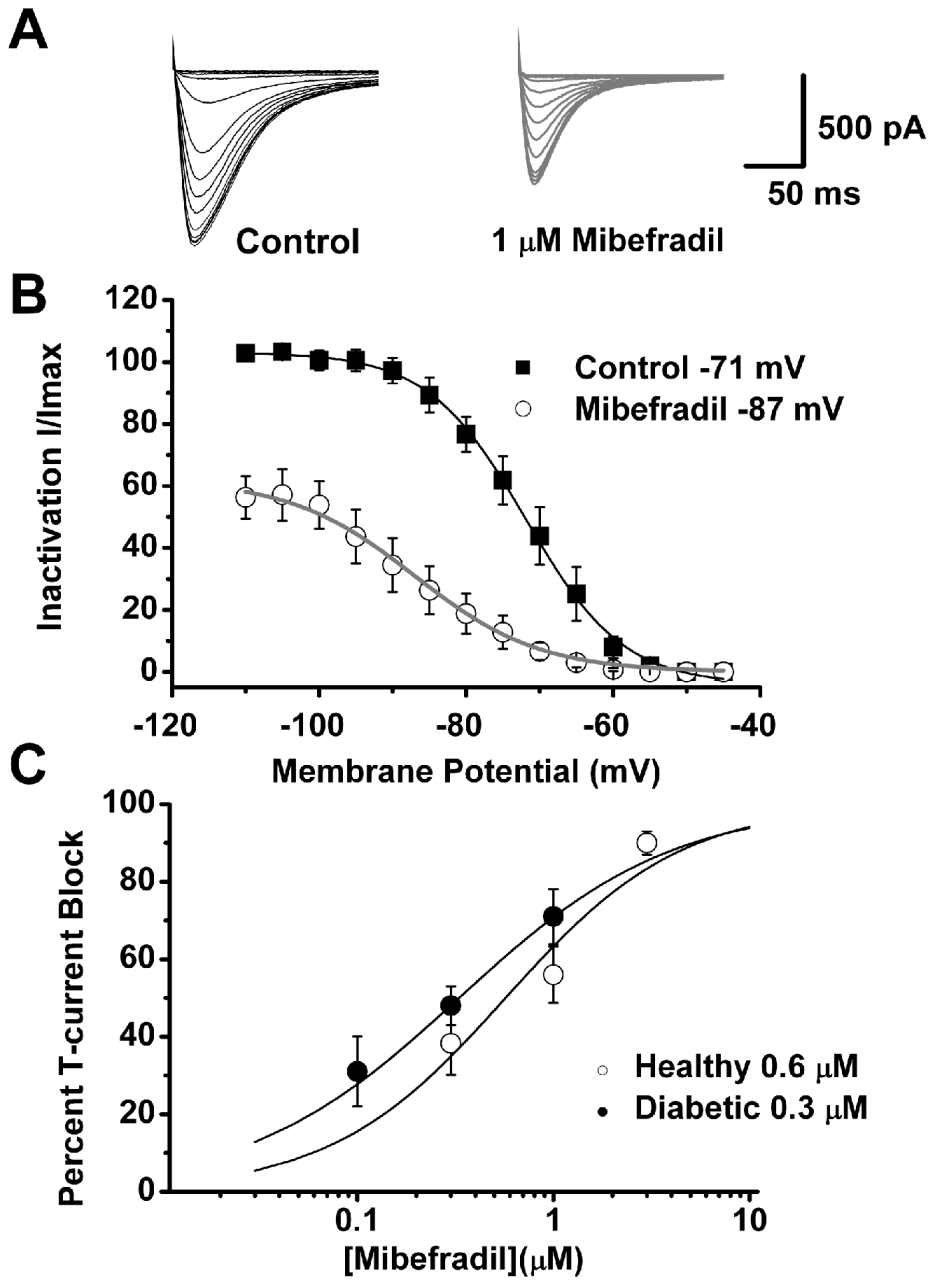
Voltage-dependent blockade of DRG T-currents in diabetic rats by mibefradil. A: Original traces from the same representative DRG cell from a diabetic rat. We used our standard double-pulse protocol (currents are evoked every 7 seconds by test steps to −40 mV after 3.5-sec pre-pulses to potentials ranging from −110 mV to −45 mV in 5 mV increments) to generate steady-state inactivation curves under control conditions (black traces) and after 5 minutes of application of 1 µM mibefradil (gray traces). Calibration bars pertain to both panels. **B**: Averaged data from 6 cells obtained before (▪) and after applications of 1 µM mibefradil (○) in the same cells. Data points are averages of multiple determinations with vertical bars representing ± SEM. Solid lines are best fits using the Boltzmann equation (2) for control (solid black line) and mibefradil (solid gray line). Peak currents are normalized to the values of T-currents at pre-pulses of −110 mV in control (predrug) conditions. Mibefradil induced a hyperpolarizing shift in steady-state inactivation curves of about 16 mV (control V_50_ −87±1 mV versus mibefradil V_50_ −71±1 mV). There was a smaller change in the slope of the curve from 6.6±0.3 mV to 8.5±0.8 mV in the presence of mibefradil. **C**: This panel summarizes the average percent inhibition of T-currents in DRG cells by escalating concentrations of mibefradil (holding potential of −90 mV and test potential of −40 mV every 7 seconds) in healthy (○) and diabetic (•) rats. Solid lines are best fits of data points using the Hill equation (1) yielding IC_50_ and slope (n) values as follows for control rats (IC_50_ = 0.6±0.2 µM, n = 1.0±0.4) and for diabetic rats (IC_50_ = 0.3±0.1 µM, n = 0.8±0.1). All fits are constrained to 100% maximal inhibition. Each data point is averaged from at least 5 different cells (control rats total of 24 cells; diabetic rats total of 20 cells). Short vertical black bars indicate ± SEM.

We have demonstrated previously that mibefradil blocks isolated DRG T-currents in healthy rats in a voltage-dependent manner [15]. To examine whether mibefradil also inhibits T-currents in a voltage-dependent manner in diabetic rats, we performed voltage-clamp experiments using acutely dissociated DRG neurons (<35 µm) (Figure 10). Representative T-current traces before and after application of 1 µM mibefradil are shown in Fig. 10A and average currents are shown in Fig. 10B. For these studies we used our standard double pulse protocols wherein pre-pulses at different voltages serve to remove inactivation of T-channels (see Materials and Methods). A fit of the average data points with a Boltzmann function (solid lines in Fig. 10B) revealed that mibefradil shifted the steady-state inactivation from a pre-drug value of −71 mV (black solid line) to −87 mV (gray solid line). We next used escalating concentrations (0.1–3 µM) of mibefradil under identical recording conditions to determine its potency in blocking DRG T-currents in healthy and diabetic rats. Fig. 10C summarizes these experiments. The solid lines are best fits of a Hill function to average data points and yield an IC_50_ for T-current inhibition in healthy rats of 0.6 µM and in diabetic rats of 0.3 µM. Even though mibefradil was somewhat more potent in blocking T-currents from diabetic than from healthy rats, there was no statistically significant difference in the amplitudes of blocked currents with 1 µM and 0.3 µM mibefradil between the two groups (Fig. 10C). Thus, we conclude that mibefradil is a more effective blocker of T-channels at depolarized membrane potential and that it inhibits with similar potency DRG T-currents in healthy and diabetic rats.

## Discussion

In this study we confirm that peripheral Ca_V_3.2 T-channels are important cellular targets for the development of drugs effective in PDN. Using *in vivo* antisense technology and pharmacological inhibition with mibefradil, we have shown that down-regulation of Ca_V_3.2 T-channels has significant therapeutic effects on the most important and the most difficult-to-treat signs of PDN – heat and mechanical hypersensitivity and cold allodynia. In addition, using *in vitro* patch-clamp recordings, we show that mibefradil inhibits T-currents in a voltage-dependent manner and with similar potency in control DRG neurons from control SAL-treated rats and DRG neurons from diabetic STZ-treated rats.

Recent studies have implicated pharmacological agents that target Ca_V_3.2 T-channels as important regulators of the cellular excitability of nociceptors and have suggested their usefulness in treating neuropathic pain in different animal models (reviewed in [22]). One such drug, mibefradil, has been used extensively for functional studies of T-channels including studies of their role in nociceptive transmission. Mibefradil was marketed by Hoffmann-LaRoche primarily as a peripherally acting antihypertensive drug. We studied mibefradil since, to our knowledge, it is the only peripherally-acting T-channel blocker that is widely available. Mibefradil has been shown to block preferentially T-currents at low micro- and nanomolar concentrations in vascular smooth muscle [23], [24] and cerebellar Purkinje cells [25]. Hence, it was considered, for a while, to be a promising selective and potent T-channel blocker. Further studies have shown that mibefradil exhibits voltage- and use-dependent inhibition of T-currents in acutely dissociated small DRG neurons *in vitro*. These properties could be beneficial for its use in pain disorders, given that the drug is more active in affecting channels in depolarized and actively firing neurons [15]. Indeed, ensuing *in vivo* studies have shown that mibefradil has mild analgesic properties in healthy rats [16] and prominent antihyperalgesic properties in rats with neuropathic pain from chronic constrictive injury (CCI) of the sciatic nerve [26]. Particularly exciting was the fact that when marketed in Europe as an antihypertensive agent, mibefradil was well tolerated, presumably due to its poor penetration into the CNS – although it consequently was withdrawn from the market due to unwanted drug-drug interactions. Studies have since indicated that mibefradil blocks not only low-voltage activated-type calcium T-currents, but also high-voltage-activated (HVA)-type calcium and other voltage-gated currents (e.g., I_Na+_ and I_K+_) at low µM concentrations, thus casting doubt on its usefulness as a selective T-channel blocker [27]–[29].

The intriguing possibility that the analgesic properties of mibefradil that we and others have reported may not be related to its blockade of T-channels in peripheral nociceptors must be raised, since its selectivity in blocking T-channels in DRG neurons is questionable. However, several lines of evidence in our present study strongly suggest that the analgesic actions of mibefradil are, indeed, mediated by peripheral T-channel blockade. First, we show that the antihyperalgesic effect of mibefradil in PDN was fully mimicked by knock-down of Ca_V_3.2 channels in DRG cells using specific AS; second, we show that in animals pretreated with Ca_V_3.2 AS, mibefradil was completely ineffective in further affecting thermal and mechanical sensitivities, while its antihyperalgesic properties in Ca_V_3.2 MIS animals was maintained, thus suggesting that mibefradil shares the same cellular target as Ca_V_3.2 AS. In contrast, we show that the anti-hyperalgesic effect of morphine, a prototype opioid analgesic, was not affected by Ca_V_3.2 AS or MIS pretreatment. Consistent with these results, it has been shown that T-currents in small DRG cells are insensitive to opiod agonists [30] and that the response of Cav3.2 knock-out mice to morphine was essentially identical to that of wild type mice [31]. Since mibefradil poorly penetrates the CNS and its effects are mostly confined to peripheral targets [23], [24], its use is not known to cause the sedation that is often encountered with pain killers commonly used in PDN (such as gabapentin and related drugs) [32], [33]. Indeed, we have demonstrated previously that systemic administration of mibefradil at 9 mg/kg i.p. did not affect sensorimotor performance of rats [16]. Hence, we suggest that peripheral analogs of mibefradil that are more selective and perhaps more potent should be developed since they may prove useful not only in the functional studies of T-channels, but in the development of clinical analgesics for patients with painful PDN and other chronic pain disorders. Collectively, our data strongly suggest that Ca_V_3.2 channels in sensory neurons are important target for the prominent analgesic effects of mibefradil. However, the possible contribution of other ion channels to its analgesic effects cannot be excluded. Future biophysical and molecular studies employing knock-down of other nociceptive ion channels in DRG cells from diabetic rats will be needed to address this issue.

Another important novel finding of this study is that both mibefradil treatment and Ca_V_3.2 AS, but not morphine, greatly attenuated cold allodynia scores in diabetic rats. Similar to our observations with heat hyperalgesia and mechanical hypersensitivity, mibefradil failed to modulate cold allodynia scores in diabetic animals pretreated with Ca_V_3.2 AS but it was still effective in animals pretreated with Ca_V_3.2 MIS, further suggesting that mibefradil and Ca_V_3.2 AS share the same cellular target. This finding also points at T-channels as a promising cellular target for treating hypersensitivity to cold stimuli in patients with PDN. Cold allodynia is a common sign of neuropathic pain in patients and in animal models of PDN [20], [21] but its underlying mechanisms remain poorly understood. This poor understanding is, at least in part, due to the fact that ion channels involved in cold transduction are not fully characterized. Recent data with agents targeting transient receptor potential (TRP) channels (especially TRPA1 and TRPM8) suggest that these channels are promising targets in developing treatments for cold allodynia [21]. However, very little is known about the role of T-channels in cold transduction pathways in the context of PDN. *In vitro* studies with small DRG neurons have shown that cold, similarly to heat, evokes inward currents in sensory neurons such that the somas fire multiple action potentials, which resemble burst firing [33]. This multiple firing is relevant to our study since T-channels are key regulators of cellular excitability and burst firing in small DRG neurons [34], [35]. Although T-currents have been recorded in cold-sensitive sensory neurons [36], their role in excitability of cold-sensitive neurons has not been studied. Our *in vivo* data strongly suggest a supportive role of T-channels in cold-induced neuronal transduction. Further cellular and molecular studies are needed to investigate this notion, especially since T-channel blockers may be very effective in treating cold allodynia in diabetic patients, a debilitating condition very refractive to conventional treatment.

In conclusion, our data indicate that blocking T-channels with the peripherally-acting, voltage-dependent agent mibefradil strongly attenuates diabetes-induced heat, cold and mechanical hypersensitivity in STZ-treated rats. These results suggest that further experimental and clinical studies can open avenues for the pharmacological development of novel and more specific therapies targeting ion channels in peripheral nociceptors. This approach could be useful for pain control in patients with diabetic neuropathy while minimizing side effects.
